# Investigating expressed RNA variants that are related to disease severity in SARS-CoV-2-infected patients with mild-to-severe disease

**DOI:** 10.1186/s43042-022-00299-5

**Published:** 2022-04-28

**Authors:** Javan Okendo, David Okanda

**Affiliations:** 1 Systems and Chemical Biology Division, Department of Integrative Biomedical Sciences, Institute of Infectious Disease and Molecular Medicine, Faculty of Health Sciences, University of Cape Town, Anzio Road Observatory, Cape Town, 7925 South Africa; 2 Research, Innovations, and Academics Unit, Tunacare Services Health Providers Limited, Nairobi, Kenya

**Keywords:** RNAseq, Variants, SARS-CoV-2, Severe, ICU, Moderate

## Abstract

**Background:**

Severe acute respiratory syndrome *coronavirus 2 *(SARS-CoV-2) continues to be a significant public health challenge globally. SARS-CoV-2 is a novel virus, and the understanding of what constitutes expressed RNAseq variants in healthy, convalescent, severe, moderate, and those admitted to the intensive care unit (ICU) is yet to be presented. We characterize the different expressed RNAseq variants in healthy, severe, moderate, ICU, and convalescent individuals.

**Materials and methods:**

The bulk RNA sequencing data with identifier PRJNA639275 were downloaded from Sequence Reads Archive (SRA). The individuals were divided into: (1) healthy, *n* = 34, moderate, *n* = 8, convalescent, *n* = 2, severe, *n* = 16, and ICU, *n* = 8. Fastqc version 0.11.9 and Cutadapt version 3.7 were used to assess the read quality and perform adapter trimming, respectively. STAR was used to align reads to the reference genome, and GATK best practice was followed to call variants using the rnavar pipeline, part of the nf-core pipelines.

**Results:**

Our analysis demonstrated that different sets of unique RNAseq variants characterize convalescent, moderate, severe, and those admitted to the ICU. The data show that the individuals who recover from SARS-CoV-2 infection have the same set of expressed variants as the healthy controls. We showed that the healthy and SARS-CoV-2-infected individuals display different sets of expressed variants characteristic of the patient phenotype.

**Conclusion:**

The individuals with severe, moderate, those admitted to the ICU, and convalescent display a unique set of variants. The findings in this study will inform the test kit development and SARS-CoV-2 patients classification to enhance the management and control of SARS-CoV-2 infection in our population.

## Introduction

Severe acute respiratory syndrome coronavirus 2 (SARS-CoV-2) infections remain a global public health challenge [1]. The SARS-CoV-2 infections continue to take an upward trajectory, and as of February 20, 2022, there were 5.8 million confirmed deaths and an excess of 415 million confirmed cases globally (https://coronavirus.jhu.edu/map.html). Coronavirus disease 2019 (COVID-19) spreads from person to person through direct contact or encountering infected surfaces [2]. When SARS-CoV-2 is inhaled, it enters the human host cells via angiotensin-converting enzyme 2 (ACE2) receptors [3]. Once the virus enters the human cells, it starts replicating, leading to population expansion within the cells [3], while in the cells, it induces the local immune cells to start producing cytokines and chemokines, resulting in the attraction of other immune cells in the lung, which causes excessive tissue damage [4]. A growing body of evidence indicates that the SARS-CoV-2 virus is not confined to the human lungs [1]. Still, it also affects the other body organs, such as the kidney, where it causes acute kidney injury (AKI) [1, 5]. In other individuals infected with SARS-CoV-2, neurological, cardiovascular, and intestinal malfunctions have also been reported [6].

SARS-CoV-2 continues to evolve, resulting in the emergence of different variants with varying degrees of virulence [7]. Genomic investigations have been integral in SARS-CoV-2 surveillance; for example, the Network for Genomic Surveillance South Africa (NGS-SA) consortium has been at the forefront in real-time tracking of this rapidly mutating virus [8]. The inherent mutational ability of SARS-CoV-2 has led to multiple variants classified into four groups: variants of concern (VOC), variants of interest (VOI), variants being monitored (VBM), and variants of high consequence (VOHC) (www.cdc.gov). The SARS-CoV-2 variants are further classified by using the letters of the Greek alphabet, e.g., Alpha, Beta, Delta Gamma, Iota, Kappa, Lambda, Omicron, etc., for easy-to-say labeling (www.who.int). Currently, three VBMs (Alpha-B.1.1.7, Beta-B.1.351, and Gamma-P.1) and two VOCs (Delta-B.617.2 including AY sub-lineages and Omicron-B.1.1.529 including BA lineages) are in circulation worldwide (www.cdc.gov). The Omicron variant has predominated over other variants globally [9].

Understanding the expressed variants underlying a broad spectrum of SARS-CoV-2 presentation is a fundamental step. Characterizing the expressed variants will help us understand what constitutes the differential manifestation of SARS-CoV-2 in our population and how to manage this pandemic. Studies have been conducted to characterize the SARS-CoV-2 variant using SARS-CoV-2 whole genomes sequences, which have aided the identification of single-nucleotide polymorphisms, insertions and deletions, and structural variants [10]. Structural bioinformatics has also been used to identify the effects of SARS-CoV-2 mutations on the native structure of the S-protein of SARS-CoV-2 by studying the D614G mutation [11]. In another related study, the impact of SARS-CoV-2 on the human host was investigated. It was demonstrated that SARS-CoV-2 infection increased the expression of angiotensin-converting enzyme 2 (ACE2) in the pancreatic islet cells in diabetic donors [12]. This study used the bulk RNAseq variant calling approach to study the expressed variants from individuals with different clinical outcomes post-SARS-CoV-2 infections. The findings in this study will provide a list of variants with the corresponding genes which can inform drug discovery and development research.

## Materials and methods

### Study samples description

The study participants were divided into gender, male, *n* = 36, and female, *n* = 32. To gain more insight into the SARS-CoV-2 disease, the individuals were further grouped depending on the severity of SAR-CoV-2 infection, healthy individuals, *n* = 34, moderate infection, *n* = 8, convalescent, *n* = 2, severe cases, *n* = 16, and individuals in the intensive care unit (ICU), *n* = 8. The individuals who had a confirmed negative for viral RNA polymerase chain reaction (PCR) were considered healthy, while those with confirmed positive PCR results were considered infected. Further details on the peripheral blood mononuclear cells (PBMCs) preparation protocols and detailed patient characteristics have been reported in the literature [13].

### RNA sequencing variants calling

The preprocessing of the Fastq files was conducted using FastQC version 0.11.9 [14]. Trim galore, a wrapper around Cutadapt version 3.7 and FastQC, was used for the adapter trimming and further quality assessment of the raw file [15]. The STAR, the splice aware genome aligner, was used to align adapter-trimmed single-end reads to the human reference genome [hg38] [16]. The alignment post-processing was then conducted using the Picard tool (https://broadinstitute.github.io/picard/) with the “Picard markDuplicates” command to mark duplicate reads. Splitting reads that contain Ns in their cigar string was done using Genome Analysis Tool Kit 4 (GATK4) [17] using the “GATK4 SplitNCigarReads” function. The GATK4 Base Quality Recalibration (BSQR) was then done on the aligned reads. Calling single-nucleotide polymorphisms (SNPs) and insertions and deletions (indels) via local re-assembly of haplotypes was conducted using the “GATK4 HaplotypeCaller” function. The identified variants were further filtered using the “GATK4 VariantFiltration” command. Finally, the overall quality of the alignment and the data, in general, was assessed using MultiQC software [18]. The reported variants were then annotated to study their effects on proteins and genes using the variant effect predictor (VEP) tool [19], using “homo_sapiens” as the target organism. All these analyses were conducted using the rnavar (https://github.com/nf-core/rnavar), part of the nf-core pipelines [20]. The annotation of the identified SNPs was performed using the SNPsnap tool [21]. Downstream data analysis and visualization were conducted in the R programming language.

## Results

In this research, we hypothesize that the SARS-CoV-2 infections result in the expression of different RNA variants. We analyzed bulk RNA sequencing data obtained from healthy, convalescent, moderately infected individuals, and severe individuals admitted to the ICU in different health facilities in Atlanta, Georgia, USA. The host RNA variants expression was assessed to gain more insight into what constitutes SARS-CoV-2 infection and pathogenesis.

### SARS-CoV-2-infected individuals clustered according to disease status

**Fig. 1:**
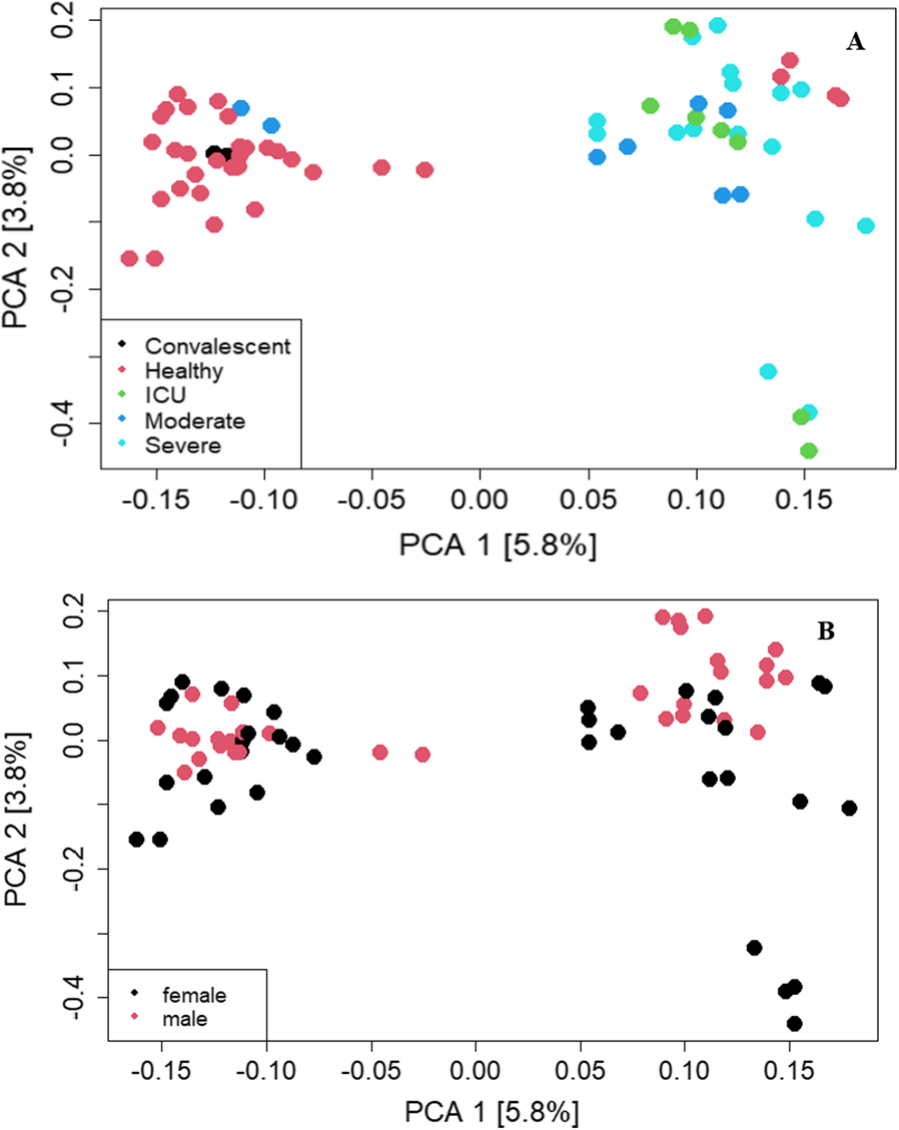
Principal component analysis showing segregation of individuals in our study. **A** The segregation of healthy, convalescent, ICU, moderate, and severe. The corresponding disease is indicated with the color code in the figure key. **B** The clustering of male and female individuals in our study

A recent study using multi-omics approaches such as proteomics, transcriptomes phosphoproteome, and ubiquitinome demonstrated that SARS-CoV-2 infections cause perturbations of the host upon infection at different omics levels [22]. Following SARS-CoV-2 infections in human hosts, it has been demonstrated that it affects different body sites such as epithelium layers, kidneys, enterocytes, and lung injuries [5]. To this end, we wondered whether the expressed RNA variants can be used to gain more insight into the pathogenesis of SARS-CoV-2 using bulk RNAseq data from healthy (*n* = 34), convalescent (*n* = 2), ICU (*n* = 8), moderate (*n* = 8), and severe (*n* = 16) SARS-CoV-2-infected individuals. According to (Fig. 1A), healthy individuals have different expressed RNA variants compared with SARS-CoV-2-infected individuals regardless of the SARS-CoV-2 infection status. Interestingly, our data demonstrate that the convalescent individuals cluster together with the healthy individuals. The data indicate heterogeneity because some healthy individuals clustered together with SARS-CoV-2-infected individuals. The clustering of healthy individuals with the SARS-CoV-2 individuals can be due to false-negative results that wrongly classified these individuals as healthy. They were already infected but remain asymptomatic (Fig. 1A). Another plausible explanation could be because once an individual recovers from SARS-CoV-2 infection, the transcription of gene variants involved in the response to infection stops. Our analysis reveals that the SARS-CoV-2 disease state does not have an impact on the expressed RNA variants in individuals with severe, moderate, and those in the ICU. In Fig. 1B, our analysis demonstrated that the response to SARS-CoV-2 infection is the same in male and female patients and indication that the expressed variants are the same in male and female patients.

### The SARS-CoV-2 disease state is characterized by the different abundance of expressed gene variants

**Fig. 2:**
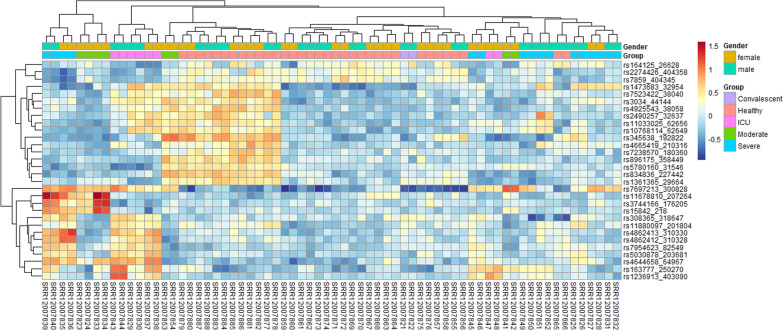
Heatmap showing the variant expression in healthy, convalescent, ICU, moderately, and severely SARS-CoV-2-infected patients in our study cohorts

We investigated the relative abundance of the expressed RNA variant across the five cohorts we compared in our analysis. There is a clear distinction in the abundance of expressed RNA variants in the healthy and SARS-CoV-2-infected individuals (Fig. 2). Our study shows that the RNA variants in ICU, severe, and moderately infected individuals have the same abundant expressed RNA variant post-SARS-CoV-2 infection (Fig. 2). An indication that the expressed RNAseq variants remain the same in the aforementioned patient cohort even though the degree of severity differs. The convalescent and healthy individuals cluster together, indicating that their transcriptomic profile reverts to the healthy status once the individuals recover from SARS-CoV-2 infection. However, the expressed RNA variants in the severe patients' cohorts demonstrated a unique pattern in their expression profile. Some variants were more abundant in one group of severely infected individuals and less abundant in another group (Fig. 2). The analysis shows that the variant expression following SARS-CoV-2 infection is not different in male and female patients.

### SARS-CoV-2-infected individuals are characterized by unique sets of RNAseq variants

**Fig. 3:**
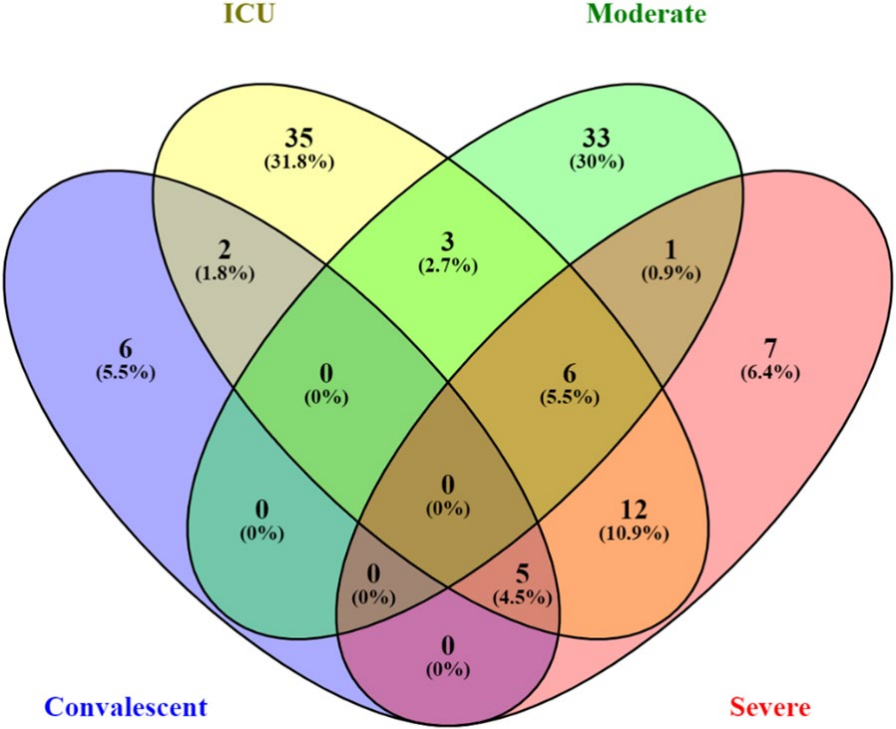
Venn diagram showing the distribution of unique variants in the convalescent, ICU, moderately, and severely SARS-CoV-2-infected individuals compared to the healthy control individuals. “Blue color” denotes convalescent, “yellow color” denotes ICU, “green color” denotes moderate, and “red color” denotes severe cases of SARS-CoV-2 infection

SARS-CoV-2 manifests differently in different individuals, resulting in severe, moderate, and critical clinical manifestations that require ICU admission and the convalescent groups [13]. Our analysis demonstrates that these groups of patients have different sets of unique SNP variants which characterize these patients. Comparing each patient cohort to the healthy controls, we identified unique sets of variants in convalescent, ICU, moderately, and severely infected individuals (Fig. 3). In convalescent individuals, we identified 6 (505%) unique variants (Fig. 3 and Table 1). Individuals admitted to the ICU had the highest number of unique variants, 35 (31.8%), followed by moderately infected individuals, 33 (30%), and the severely infected individuals had 7 (6.4%) unique RNAseq variants in Table 1 and Fig. 3. Interestingly, the ICU and severity infected individuals had the highest expressed variants overlap and indicated that the expressed SNP variants in the ICU also characterize the severely infected individuals (Fig. 3).

**Table 1:** The unique SNPs which characterize convalescent, ICU, moderately, and severely SARS-CoV-2-infected individuals when compared against healthy control individuals in our analysis

Convalescent	ICU	Moderate	Severe
rs4925543	rs5773124	rs426336	rs4919633
rs11250238	rs2810904	rs7523422	rs2957516
rs6597801	rs947480	rs2778979	rs937540
rs60754073	rs5780160	rs945310	rs2627222
rs7335867	rs2782828	rs945309	rs8106384
rs568078	rs708776	rs421262	rs6736913
	rs1017361	rs11071466	rs2721167
	rs6602492	rs2053632	
	rs7081726	rs1650996	
	rs637186	rs1230402	
	rs949324	rs1230403	
	rs949323	rs6431959	
	rs4944853	rs7593113	
	rs6589689	rs2695342	
	rs3759301	rs6042507	
	rs395545	rs2249317	
	rs27194	rs6045554	
	rs1599882	rs444271	
	rs7249826	rs713942	
	rs6083718	rs910798	
	rs4838864	rs1042979	
	rs1533270	rs10582753	
	rs6763154	rs3087885	
	rs4689037	rs4862412	
	rs4689524	rs832582	
	rs758328	rs67794491	
	rs10028164	rs308365	
	rs1291602	rs28376231	
	rs40273	rs4895944	
	rs2234026	rs614754	
	rs1546963	rs327222	
	rs3747807	rs7854035	
	rs2442513	rs7039828	
	rs2231527		
	rs1044531		

## Discussion

Multi-omics approaches have been employed to understand the pathogenesis and immune response following SARS-CoV-2 infections in humans [23–25]. These studies have been conducted to understand the main biomarkers and potential drug targets to control the spread of the novel SARS-CoV-2 virus globally. The clinical manifestations of SARS-CoV-2 infection include fever, headache, cough, muscle pain, diarrhea, and myocarditis, among others [26]. Previous studies have demonstrated that the muscle pains that characterize SARS-CoV-2 infection in humans are caused by the cytokine storm [27]. The primary source of cytokine in the infected individuals is the infected macrophages and the lung epithelial cells [27]. We demonstrated that the individuals infected with SARS-CoV-2, a novel coronavirus, cluster distinctly from the healthy control. Some healthy individuals overlapped into the disease cohort. This can be plausibly explained due to the false-negative results that categorized the individuals as healthy and yet asymptomatic. The convalescent individuals showed that after recovery from the infection, the expression of the RNAseq variants reverts to normal hence the clustering together between the healthy and the convalescent patient groups. The moderately, severely, and ICU patients did not cluster tightly together. This can be attributed to the angiotensin-converting enzyme 2 (ACE2) differential expression in different patient conditions we compared, hence the differential immune response following SARS-CoV-2 infections in humans [28]. The expressed immune variants post-SARS-CoV-2 infection is similar in female and male patient cohorts as was demonstrated in our data. The similarity in the immune response is attributed to the angiotensin-converting enzyme 2 (ACE2), and SARS-CoV-2 entry point into the human cells [29] is expressed in the lungs, blood vessels, kidneys, liver, and gastrointestinal tract in male and female patients [30].

The entry of the SARS-CoV-2 virus into the human cell is facilitated by SARS-CoV-2 spike proteins binding to the ACE2 receptor of the host membrane [31]. The clinical manifestation of SARS-CoV-2 post-infection ranges from moderate to severe, requiring ICU admission [27]. The broad spectrum of SARS-CoV-2 infection (moderate, convalescent, severe, and ICU) is attributed to the difference in the expression profiles of ACE2 in different individuals [32]. Our analysis demonstrates that individuals with severe and ICU admissions cluster together, indicating that the variant expression levels in this patient cohort are the same. Celik et al. 2021 demonstrated that the expression levels of the ACE2 genes did not differ in mild, moderately, and severely SARS-CoV-2-infected individuals [33]. In an attempt to understand the immune response to SARS-CoV-2 infection in male and female individuals, a study [30] found that the ACE2 expression in males and females differed. In our data, the expressed variants indicate that the immune response post-SARS-CoV-2 infection in males and females is the same. There must be some hitherto undiscovered immune dynamics that require further research to help us gain more insight into this unique observation.

The expressed RNA variants in severe, moderate, and ICU patients showed similar abundance levels in this patient cohort. A clear indication that there are some intrinsic dynamics at the patient level can be attributed to the SARS-CoV-2 degree of outcome in patients [30]. Interestingly, the healthy individuals showed a distinct clustering and indication that SARS-CoV-2 infection indeed causes a shift in the profiles of expressed variants. The relative abundance analysis demonstrated that the more abundant expressed RNAseq variants were less abundant in the control and the convalescent individuals. Interestingly, some severely sick individuals showed the downregulation of the expressed variants, which were more abundant in some patients in the group. This difference can be due to heterogeneity in response to an infection in a population attributed to variants in the human leucocyte antigen (HLA) gene [6].

Convalescent, moderate, severe, and individuals admitted to the ICU facilities following SARS-CoV-2 infection have a unique set of expressed RNA variants. An indication that these individuals will require different management in the health facilities. We opine that these unique sets of variants can be added to the list of biomarkers that can be used to classify individuals at testing facilities around the world.

## Conclusion

Our study demonstrated that the expressed RNAseq variants in individuals infected with SARS-CoV-2 are different. This is a proof-of-concept study demonstrating that SARS-CoV-2 therapeutics and drugs should be designed to target a specific group of patients depending on the disease severity. We showed that individuals infected with SARS-CoV-2 harbor a different set of unique expressed RNAseq variants, which act as a potential drug target. The SNPs can be used to assess the response to the currently used intervention methods and prognosis in the future.

## Data Availability

The bulk RNAseq data sets used in this study are available on SRA with PRJNA639275 identifier.

## Abbreviations

ACE2: Angiotensin-converting enzyme 2
AKI: Acute kidney injury
GATK4: Genome analysis toolkit 4
ICU: Intensive care unit
NGS-SA: Network for genomic surveillance South Africa
PCA: Principal component analysis
RNA: Ribonucleic acid
SARS-CoV-2: Severe acute respiratory syndrome coronavirus *2*
SNPs: Single-nucleotide polymorphisms
SRA: Sequence reads archive
STAR: Spliced transcripts *Alignment* to a reference
VBM: Variants being monitored
VEP: Variant effect predictor
VOC: Variants of concern
VOI: Variants of interest

## References

[1] Diamond MS, Kanneganti T-D (2022) Innate immunity: the first line of defense against SARS-CoV-2. Nat Immunol 23(2):165–176 35105981 10.1038/s41590-021-01091-0 PMC8935980

[2] Campos DMO, Fulco UL, de Oliveira CBS, Oliveira JIN (2020) SARS-CoV-2 virus infection: targets and antiviral pharmacological strategies. J Evid Based Med 13(4):255–260 33058394 10.1111/jebm.12414 PMC7675315

[3] Hoffmann M, Kleine-Weber H, Schroeder S, Krüger N, Herrler T, Erichsen S et al (2020) SARS-CoV-2 cell entry depends on ACE2 and TMPRSS2 and is blocked by a clinically proven protease inhibitor. Cell 181(2):271-280.e8 32142651 10.1016/j.cell.2020.02.052 PMC7102627

[4] Trypsteen W, Van Cleemput J, van Snippenberg W, Gerlo S, Vandekerckhove L (2020) On the whereabouts of SARS-CoV-2 in the human body: a systematic review. PLoS Pathog 16(10):1–26. 10.1371/journal.ppat.1009037 10.1371/journal.ppat.1009037 PMC7679000 33125439

[5] Joseph A, Zafrani L, Mabrouki A, Azoulay E, Darmon M (2020) Acute kidney injury in patients with SARS-CoV-2 infection. Ann Intensive Care. 10.1186/s13613-020-00734-z 32880774 10.1186/s13613-020-00734-z PMC7471244

[6] Huang C, Wang Y, Li X, Ren L, Zhao J, Hu Y et al (2020) Clinical features of patients infected with 2019 novel coronavirus in Wuhan, China. Lancet 395(10223):497–506 31986264 10.1016/S0140-6736(20)30183-5 PMC7159299

[7] Karim SSA (2021) Correspondence new SARS-CoV-2 variants—clinical, public health, and vaccine implications. N Engl J Med 384:1–3 33761203 10.1056/NEJMc2100362 PMC8008749

[8] Technologies N, Torrent I (2020) Comment A genomics network established to respond rapidly to public health threats in South Africa. Lancet 1:229–230 10.1016/S2666-5247(20)30116-6 PMC7434425 32838349

[9] Viana R, Moyo S, Amoako DG, Tegally H, Scheepers C, Althaus CL et al (2022) Rapid epidemic expansion of the SARS-CoV-2 Omicron variant in southern Africa. Nature 603:679–686 35042229 10.1038/s41586-022-04411-y PMC8942855

[10] Rouchka EC, Chariker JH, Chung D (2020) Variant analysis of 1,040 SARS-CoV-2 genomes. PLoS ONE 15(11):495–504 10.1371/journal.pone.0241535 PMC7643988 33152019

[11] Yurkovetskiy L, Wang X, Pascal KE, Tomkins-Tinch C, Nyalile TP, Wang Y et al (2020) Structural and functional analysis of the D614G SARS-CoV-2 spike protein variant. Cell 183(3):739-751.e8. 10.1016/j.cell.2020.09.032 32991842 10.1016/j.cell.2020.09.032 PMC7492024

[12] Taneera J, El-huneidi W, Hamad M, Mohammed AK, Elaraby E, Hachim MY (2020) Expression profile of SARS-CoV-2 host receptors in human pancreatic islets revealed upregulation of ACE2 in diabetic donors. Biology (Basel) 9(8):1–10 10.3390/biology9080215 PMC7465557 32784802

[13] Arunachalam PS, Wimmers F, Mok CKP, Perera RAPM, Scott M, Hagan T et al (2020) Systems biological assessment of immunity to mild versus severe COVID-19 infection in humans. Science (80-) 369(6508):1210–1220 10.1126/science.abc6261 PMC7665312 32788292

[14] Andrews S (2010) FastQC. Babraham Bioinforma

[15] Martin M (2011) Cutadapt removes adapter sequences from high-throughput sequencing reads. EMBnetjournal 17:1–10

[16] Rosenbloom KR, Armstrong J, Barber GP, Casper J, Clawson H, Diekhans M et al (2015) The UCSC genome browser database: 2015 update. Nucleic Acids Res 43:D670–D681 25428374 10.1093/nar/gku1177 PMC4383971

[17] McKenna A, Hanna M, Banks E, Sivachenko A, Cibulskis K, Kernytsky A, Garimella K, Altshuler D, Gabriel S, Daly M, DePristo MA (2010) The Genome analysis toolkit: a MapReduce framework for analyzing next-generation DNA sequencing data. Genome Res 20:1297–1303 20644199 10.1101/gr.107524.110 PMC2928508

[18] Ewels P, Magnusson M, Lundin S, Käller M (2016) MultiQC: Summarize analysis results for multiple tools and samples in a single report. Bioinformatics 32:3047–3048 27312411 10.1093/bioinformatics/btw354 PMC5039924

[19] McLaren W, Gil L, Hunt SE, Riat HS, Ritchie GRS, Thormann A et al (2016) The ensembl variant effect predictor. Genome Biol 17(1):1–14. 10.1186/s13059-016-0974-4 27268795 10.1186/s13059-016-0974-4 PMC4893825

[20] Ewels PA, Peltzer A, Fillinger S, Patel H, Alneberg J, Wilm A et al (2020) The nf-core framework for community-curated bioinformatics pipelines. Nat Biotechnol 38(3):276–278 32055031 10.1038/s41587-020-0439-x

[21] Pers TH, Timshel P, Hirschhorn JN (2015) SNPsnap: a web-based tool for identification and annotation of matched SNPs. Bioinformatics 31(3):418–420 25316677 10.1093/bioinformatics/btu655 PMC4308663

[22] Stukalov A, Girault V, Grass V, Karayel O, Bergant V, Urban C et al (2021) Multilevel proteomics reveals host perturbations by SARS-CoV-2 and SARS-CoV. Nature 594:246–252. 10.1038/s41586-021-03493-4 33845483 10.1038/s41586-021-03493-4

[23] Wang X, Xu G, Liu X, Liu Y, Zhang S, Zhang Z (2021) Multiomics: unraveling the panoramic landscapes of SARS-CoV-2 infection. Cell Mol Immunol 18(10):2313–2324. 10.1038/s41423-021-00754-0 34471261 10.1038/s41423-021-00754-0 PMC8408367

[24] Maras JS, Sharma S, Bhat A, Rooge S, Aggrawal R, Gupta E et al (2021) Multi-omics analysis of respiratory specimen characterizes baseline molecular determinants associated with SARS-CoV-2 outcome. iScience 24(8):102823. 10.1016/j.isci.2021.102823 34308298 10.1016/j.isci.2021.102823 PMC8268673

[25] Zheng J, Zhang Y, Liu Y, Baird D, Karim MA, Ghoussaini M et al (2020) Multi-omics study revealing putative drug targets of COVID-19 severity and other viral infection diseases. medRxiv 9:655

[26] Kang S, Peng W, Zhu Y, Lu S, Zhou M, Lin W et al (2020) Recent progress in understanding 2019 novel coronavirus (SARS-CoV-2) associated with human respiratory disease: detection, mechanisms and treatment. Int J Antimicrob Agents 55(5):105950. 10.1016/j.ijantimicag.2020.105950 32234465 10.1016/j.ijantimicag.2020.105950 PMC7118423

[27] Song P, Li W, Xie J, Hou Y, You C (2020) Cytokine storm induced by SARS-CoV-2. Clin Chim Acta 509:280–287. 10.1016/j.cca.2020.06.017 32531256 10.1016/j.cca.2020.06.017 PMC7283076

[28] Hamming I, Timens W, Bulthuis MLC, Lely AT, Navis GJ, van Goor H (2004) Tissue distribution of ACE2 protein, the functional receptor for SARS coronavirus. A first step in understanding SARS pathogenesis. J Pathol 203(2):631–637 15141377 10.1002/path.1570 PMC7167720

[29] Expression of ACE2, the SARS-CoV-2 receptor, and TMPRSS2 in prostate epithelial cells. 2020. 10.1016/j.eururo.2020.04.065 PMC7200365 32418620

[30] Devaux CA, Rolain JM, Raoult D (2020) ACE2 receptor polymorphism: susceptibility to SARS-CoV-2, hypertension, multi-organ failure, and COVID-19 disease outcome. J Microbiol Immunol Infect 53(3):425–435. 10.1016/j.jmii.2020.04.015 32414646 10.1016/j.jmii.2020.04.015 PMC7201239

[31] Beyerstedt S, Casaro EB, Rangel ÉB (2021) COVID-19: angiotensin-converting enzyme 2 (ACE2) expression and tissue susceptibility to SARS-CoV-2 infection. Eur J Clin Microbiol Infect Dis 40(5):905–919 33389262 10.1007/s10096-020-04138-6 PMC7778857

[32] Wan Y, Shang J, Graham R, Baric RS, Li F (2020) Receptor recognition by the novel coronavirus from wuhan: an analysis based on decade-long structural studies of SARS coronavirus. J Virol. 10.1128/JVI.00127-20 31996437 10.1128/JVI.00127-20 PMC7081895

[33] KarakaşÇelik S, ÇakmakGenç G, Pişkin N, Açikgöz B, Altinsoy B, Kurucuİşsiz B et al (2021) Polymorphisms of ACE (I/D) and ACE2 receptor gene (Rs2106809, Rs2285666) are not related to the clinical course of COVID-19: a case study. J Med Virol 93(10):5947–5952 34170561 10.1002/jmv.27160 PMC8426884

